# Topological domain states and magnetoelectric properties in multiferroic nanostructures

**DOI:** 10.1093/nsr/nwz100

**Published:** 2019-07-26

**Authors:** Guo Tian, Wenda Yang, Deyang Chen, Zhen Fan, Zhipeng Hou, Marin Alexe, Xingsen Gao

**Affiliations:** 1 Guangdong Provincial Key Laboratory of Quantum Engineering and Quantum Materials, and Institute for Advanced Materials, South China Academy of Advanced Optoelectronics, South China Normal University, Guangzhou 510006, China; 2 Department of Physics, University of Warwick, Coventry CV4 7AL, UK

**Keywords:** topological defects, ferroelectric domains, multiferroic nanostructures, magnetoelectric coupling

## Abstract

Multiferroic nanostructures have been attracting tremendous attention over the past decade, due to their rich cross-coupling effects and prospective electronic applications. In particular, the emergence of some exotic phenomena in size-confined multiferroic systems, including topological domain states such as vortices, center domains, and skyrmion bubble domains, has opened a new avenue to a number of intriguing physical properties and functionalities, and thus underpins a wide range of applications in future nanoelectronic devices. It is also highly appreciated that nano-domain engineering provides a pathway to control the magnetoelectric properties, which is promising for future energy-efficient spintronic devices. In recent years, this field, still in its infancy, has witnessed a rapid development and a number of challenges too. In this article, we shall review the recent advances in the emergent domain-related exotic phenomena in multiferroic nanostructures. Specific attention is paid to the topological domain structures and related novel physical behaviors as well as the electric-field-driven magnetic switching via domain engineering. This review will end with a discussion of future challenges and potential directions.

## INTRODUCTION

Multiferroic materials that possess more than one ferroic order have attracted massive interest in the past two decades, mainly because they are ideal playgrounds for exploring emergent cross-coupling phenomena among spin, charge, orbit, and lattice in correlated electron systems, as well as promising candidates for prospective applications in advanced industries, e.g. data memory/processing, sensors, actuators, and energy-relevant devices [1–10]. For instance, ferroelectric or magnetoelectric (ME) random access memories (FeRAMs/MeRAMs) are relatively simple devices each usually consisting of a transistor and a ferroelectric/multiferroic capacitor [11,12]. Once these memories scale up to a few Gbit/in^2^ in density, the characteristic dimensions of functional multiferroic nanocapacitors will shrink down to ∼10^2^ nm or less. Due to the substantial size and surface effects, the domain structures in these multiferroic components can be greatly changed, and classical domain patterns may be replaced by some unique complex domain structures [13]. Since it is known that the physical properties of ferroic materials are intimately related to the domain structures, a targeted or pre-designed reduction in the characteristic scale may efficiently tailor the domain structure and thus related functionalities, noting that previous investigations in this area have unveiled a plethora of exotic physical phenomena over the past decade.

One exciting outcome of ferroelectric size shrinkage is the potential for stabilizing various polar topological structures. It has been predicted that a bi-stable flux-closure polar vortex (a swirl polarization configuration) as small as 3.2 nm could exist in ferroelectric nanodots, promising for ultrahigh-density memories with areal density over 60 Tbit/in^2^ [14]. This prediction has spurred tremendous interest but nonetheless an experimental observation of the vortex has remained elusive for a long time. The first observation of polarization curling was reported by Jia *et al.* [15]. Soon after, flux-closure domain textures were observed in BiFeO_3_ (BFO) films, and some of them were induced by applied electric field [16–21]. Recently, atomically resolved scanning transmission electron microscopy (STEM) has enabled observation of periodic arrays of polar closure domains and vortices in PbTiO_3_/SrTiO_3_ (PTO/STO) multilayers/superlattices [22,23] and multiferroic tunnel junctions with ultrathin ferroelectric layers [24]. More recently, the polar skyrmion bubble (a topologically protected whirling texture), an analogy to magnetic skyrmions, was also uncovered in compressively strained PTO/STO superlattices [25].

On the other hand, various domain textures in well-confined nanoislands, such as closure quadrant domains (square shape flux-closure), vortex structures, and center-type domains (with polarization pointing out (or in) from (or to) a center region) have been identified in BaTiO_3_ (BTO) or BFO nanodots [26–31]. In particular, identification of electrically controllable center-type domains and the unique domain wall conduction features in small nanoislands [27–29] holds promise for implementation in novel topological memory devices utilizing current readout of topological states [28]. These tantalizing findings suggest that the size reduction in multiferroic materials may open a new avenue to a whole family of emergent physical phenomena that underpin a range of topological nanoelectronic devices.

Dimension reduction is also an effective method to amplify the ME effect in multiferroic nanostructures, in particular electric-field-driven magnetic switching (EDMS) [32–34]. Previous reports indicated that multiferroic nanocomposites such as column-matrix (1–3 type coupling) or nanodots (0–0 type coupling) may exhibit an enhanced ME coefficient or even enable EDMS (albeit rather uncontrollably) due to the release of substrate-clamping [5,33,34]. More interestingly, the controllability of EDMS can be greatly improved by optimizing the domain structures and switching dynamic modes [35–42]. To achieve high-performance EDMS that could eventually lead to practical applications such as MeRAMs, one should be able to deterministically control the domain structure and switching behaviors of both the ferroelectric and magnetic components. In addition, adopting topologically protected magnetic domains has specific advantages for EDMS [43], and is becoming very interesting recently.

In this review article, we will briefly discuss those exotic phenomena brought about by size-confined dimension reduction of multiferroic materials, specifically focusing on the topological domain structures and related ME effects. For a more general overview on ME coupling and device applications, readers may refer to several excellent review papers [8–10,12,44]. We shall start from a brief introduction to the fabrication techniques of multiferroic/ferroelectric nanostructures, and then address the topological domains and associated unique properties, followed by highlighting the recent advances in EDMS. A discussion on open questions and new directions to be explored will be presented in the final section.

## FABRICATION OF MULTIFERROIC NANOSTRUCTURES

Motivated by the fascinating functionalities and application potentials of multiferroic nanostructures, extensive efforts in fabricating high-quality nanostructures have been made [45–51]. Along this line, a number of fabrication techniques for patterning nanostructures have been developed, and these techniques can be roughly classified into top-down and bottom-up approaches, as summarized in the literature [46]. The top-down approaches, including focus ion beam milling and electron beam direct writing, enable good control of the shape and size of nanostructures but show low throughput [47]. The bottom-up approaches, including self-assembly and controlled growth methods, are able to produce large-area and high-density epitaxial nanostructures but the shape control and order arrangement may become issues [48].

**Figure 1.**
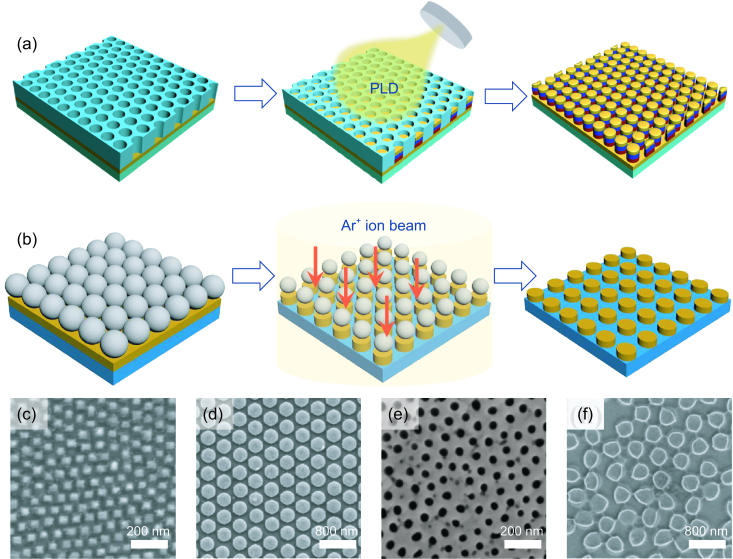
Schematic flowcharts of two representative template-assisted fabrication techniques for multiferroic nanostructures, and examples of some fabricated nanostructures. (a) The procedure of the anodized alumina (AAO) template-assisted pulsed laser deposition bottom-up method: AAO template mask transfer, material deposition, and mask removal. Reproduced with permission [33]. Copyright 2016, American Chemical Society. (b) Procedure of polystyrene (PS) nanosphere template-assisted ion beam etching method: PS template layer transfer, Ar^+^ beam etching through PS layer, and template removal. Panels (c–f) show scanning electron microscope (SEM) images of some multiferroic nanostructures fabricated via the mask-assisted methods: epitaxial nanodots (c, d), anti-nanodots (e), nanorings (f). Reproduced with permission [26], Copyright 2017, American Chemical Society; [50], Copyright 2017, Elsevier.

In this sense, modified approaches have been introduced to overcome these barriers, including template-assisted patterning, which combine the advantages of top-down and bottom-up techniques and are able to produce well-ordered and high-quality oxide nanostructures [26,49–51]. The standard procedure of a typical bottom-up anodized alumina (AAO) template-assisted pulsed laser deposition (PLD) method is shown in Fig. 1 [32,33,51] which involves three steps: AAO mask transfer, material deposition, and mask removal (see Fig. 1a). This technique is able to produce large-area and well-ordered multiferroic nanodots with diameters from ∼35 nm to ∼300 nm, and has been utilized extensively in the fabrication of multilayered nanodots, e.g. BaTiO_3_/CoFe_2_O_4_ (BTO/CFO) and BiFeO_3_/CoFe_2_O_4_/SrRuO_3_ (BFO/CFO/SRO) nanodot arrays [32,33]. Nevertheless, material deposition into the small holes of the templates requires relatively low deposition ambient pressure and temperature, and thus unwanted defects may appear easily. To avoid these defects, template-assisted top-down methods by Ar^+^ beam etching through a monolayer PS sphere array or AAO template placed on high-quality epitaxial thin film have been developed, as shown in Fig. 1b [26]. By choosing proper template geometric parameters and etching time, periodically ordered nanodots with tunable sizes (diameters over 100 nm to 900 nm) can be obtained, and the patterned nanostructures preserve their epitaxy and ferroelectric properties comparable to their parent films. Using similar modified template-assisted etching, one is able to develop high-density arrays of multiferroic nanostructures, e.g. nano-disks, nano-rings, and anti-nanodot arrays [49,50]. Other techniques like the di-block-copolymer template-assisted self-assembled method have been developed too, which allows an array of ultra-small nanodots of 10 nm in diameter [48]. These techniques lay a good foundation for further explorations into exotic physic phenomena and prospective devices. Due to length limitations, we are not going to include more discussion here and readers may refer to an earlier review for details [46].

## TOPOLOGICAL POLAR DOMAINS IN LOW-DIMENSION SYSTEMS

Ferroic domain structure is rather essential, not only for its role as data storage media in memory devices but also considering its intimate relation with a wide range of functionalities such as piezoelectricity, conductivity, magnetic exchange bias, as well as ME coupling [52,53]. Since domain structures depend critically on various interactions including exchange coupling, strain energy, electrostatic energy, and others, which, however, are highly competitive, dimension shrinkage into nanostructures becomes an effective tool to manipulate domain structures and thus relevant properties [26–31,54–59]. In particular, size-confined ferroic nanostructures favor intriguingly exotic topological domains. For example, vortex textures can be thermodynamically stabilized in nanoscale systems if the prominent depolarization effect is not well screened or compensated by other sources [14].

Currently, these topological domain structures are receiving a great deal of attention. According to Mermin, a topological defect is defined as a region with low-dimensional singularity, in which order parameter ceases to vary continuously [60]. The well-known ferroic domain wall can be classified as a 2D topological defect [61–63], while more complex domains, such as the flux-closure vortex, anti-vortex, center domain, and skyrmion, can be classified as quasi-1D topological defects. There are also other complex topological domains such as flux-closure quadrants, which do not have 1D topological cores, and thus cannot be classified as 1D defects. These exotic topological domains not only strongly determine the domain switching behaviors, but also serve as ideal arena that enable creation, nucleation, and displacement of a plethora of fantastic and localized phenomena, offering opportunities for ultrahigh-density configurable topological electronic devices [28,64–66].

Over the past decade, magnetic topological defects such as the vortex and skyrmion have been widely studied as essential building blocks for the next generation of magnetic data storage [67,68]. In contrast, investigations into complex polar defects (electric-dipole related) are still in their infancy. Although polar vortex structures were predicted long time ago, their experimental observations remain elusive, due to the lack of effective probing techniques. Thanks to the invention of powerful piezoresponse force microscopy (PFM) and advanced transmission electron microscopy (TEM), experimental evidence for non-trivial polar defects has been appearing, including recently observed ferroelectric skyrmion bubble domains [25]. Those so far experimentally identified exotic topological polar domains are summarized in Table 1, and most of them have been observed in size-confined ferroelectric/multiferroic nanostructures, e.g. ultrathin films [15,16], multilayers/superlattices [22,23,25], nanoislands [26–28,55], and single-crystal nanoplates [54,69,70]. In some cases, polar topological textures may appear in thick films or bulk systems as metastable states, e.g. a quadrupole vortex created by applying an electric field in BFO thin films [17–20], or multi-fold vortices in single crystals of improper ferroelectrics (e.g. rare-earth manganates) [71–73]. In this section, we focus on those complex polar topological defects in size-confined systems (ultrathin films/multilayers and confined nanodots/nanoislands. Readers are directed to earlier reviews on similar topics in films and bulk crystals [13,71].

**Table 1.** A schematic summary of experimentally observed exotic topological polar domains. BFO: BiFeO_3_, PZT: Pb(Zr, Ti)O_3_, STO: SrTiO_3_, PTO: PbTiO_3_, BTO: BaTiO_3_.

Topological domain type	Polarization configuration	Material and size	Functionality and prospective application
Vortex/anti-vortex	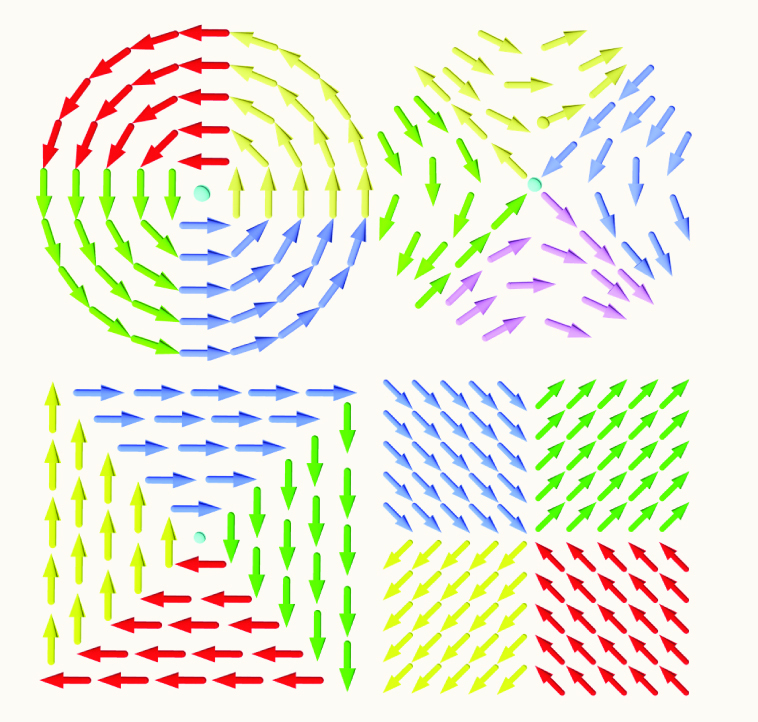	BFO nanoislands: ∼300 nm in diameter [26]; PZT nanoisland: ∼60 nm in diameter [55]; BFO films: ∼50–60 nm [17,19], ∼10 nm thick [21]; STO/PTO superlattice: 4–8 nm thick PTO [23]	Enhanced conductivity in vortex core [17]; High-density memory [19]; Negative capacitance, energy-efficient transistors [79]
Six-fold vortex/anti-vortex	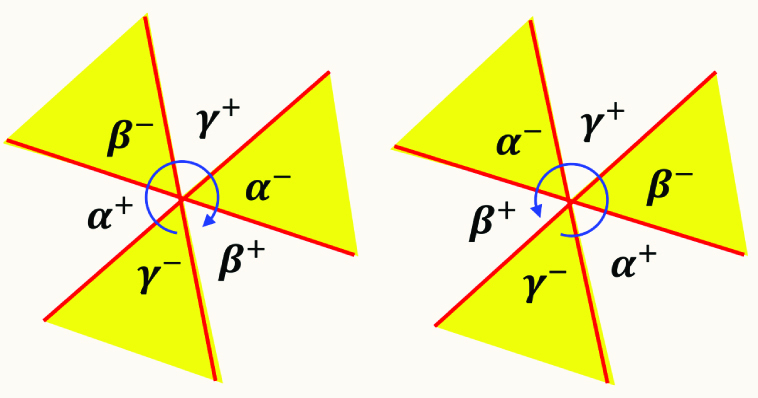	RMnO3 single crystal (R=Y, Ho, …, Lu, Sc): bulk [71]	Conductive domain walls [13]; Magnetoelectric effects [73]
Flux-closure quadrant	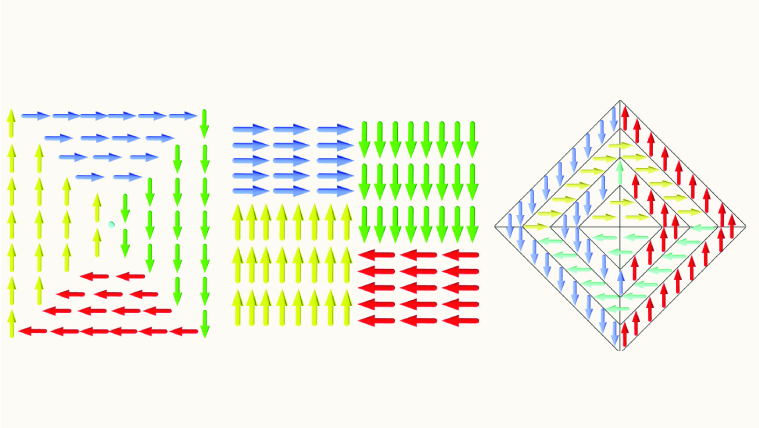	STO/PTO multilayers: ∼20–40 nm thick PTO [22]; BTO and PZT nanoplates: lateral size ∼1 μm [54,69,70]	High-density non-volatile memories [22]
Other closure domain	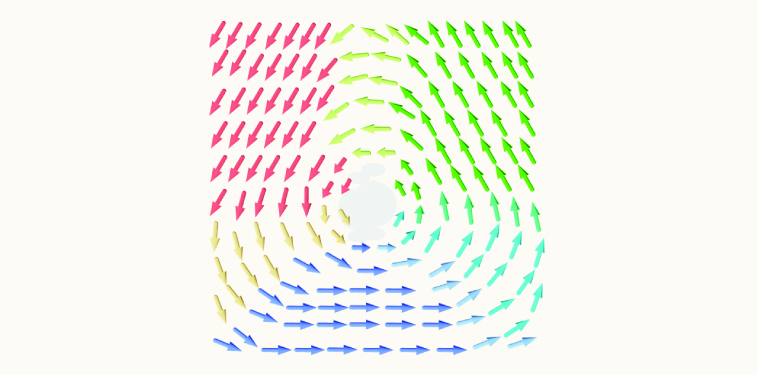	PZT films: ∼11 nm [15]; BFO films: ∼20 nm [16]	Ultrahigh-density non-volatile memories [15]
Center domain	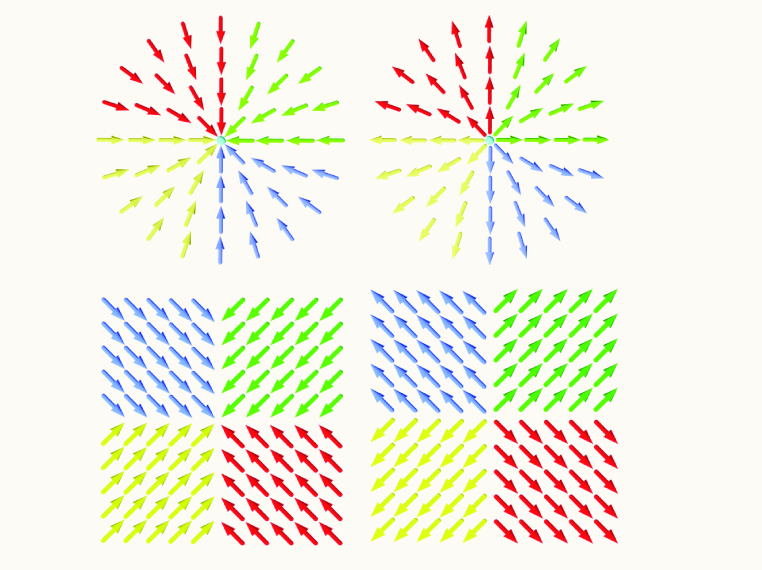	BFO nanoislands: ∼60 nm [27], ∼300 nm [28], ∼300 nm [31] in diameter; BFO thin films: ∼800 nm thick [20]	High-density non-volatile memories [27]; Conductive domain walls [28]
Skyrmion bubble	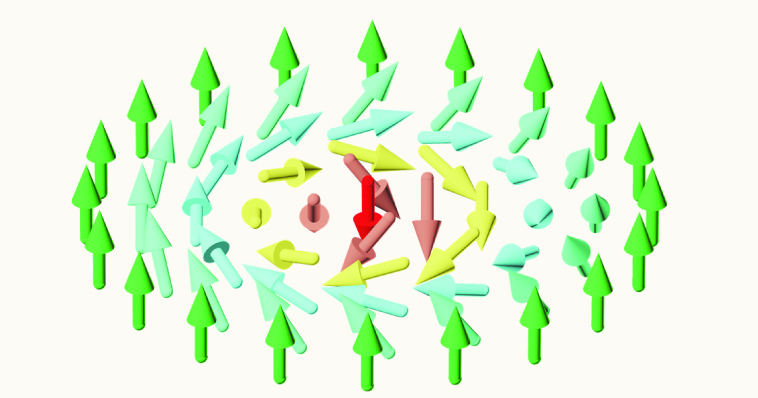	STO/PTO superlattice: 6–8 nm thick PTO [25]	Memories and other electronic nano-devices [25]

### Polar topological structures confined in ultrathin films/multilayers

Investigations on topological structures in ferroic systems have been mainly driven by the increasing demand for high-density data storage using ultra-small objects to store ‘1’ and ‘0’ data bits. One candidate is the flux-closure vortex in ferromagnetic nanostructure, predicted by Landau and Lifshitz [74] as well as Kittel [75] 70 years ago and investigated recently for the purpose of ultra high density memory devices [76]. The formation of magnetic vortices is mainly driven by large demagnetization in nanomagnets, which tends to curl the spin configuration for minimizing the total magneto-static energy. While ferroelectrics and ferromagnets have many similarities, nevertheless ferroelectric counterparts of magnetic vortex domain have remained elusive for a long time. Unlike magnetization, which can be rotated continuously to generate curling-like patterns, ferroelectric polarization is usually strongly coupled with the crystal lattice, and a high elastic energy cost is required to bend the polarization.

For ferroelectrics, it was not until 2004 that Naumov *et al.* [14] and Kornev *et al.* [77], by using first-principles simulations, predicted the emergence of a flux-closure vortex with toroidic order in nanoscale confined systems. Such toroidic-order flux-closure vortices indicate the possibility of polarization bending in a structure confined down to several nanometers (a length scale comparable with the intrinsic domain wall width), wherein the depolarization field dominates so that a curling of polarization near the interface/surface or domain wall eventually develops.

**Figure 2.**
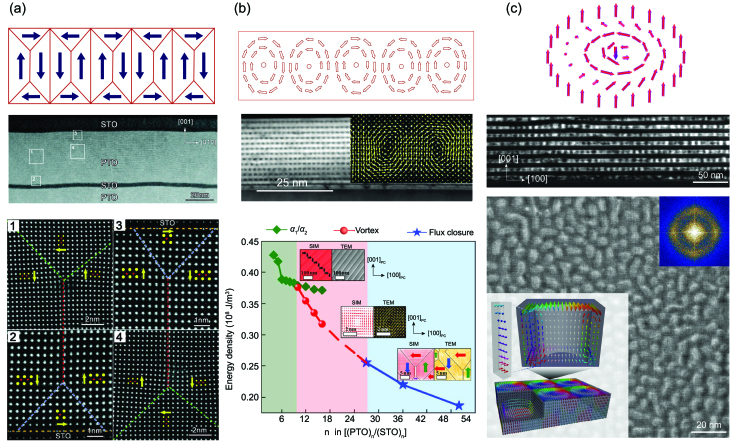
Some representative topological domains identified in SrTiO_3_/PbTiO_3_ multilayers/superlattices. (a) A flux-closure quadrant lattice in a multilayer consisting of SrTiO_3_ (10 nm)/PbTiO_3_ (36 nm)/SrTiO_3_ (3 nm)/PbTiO_3_ (28 nm)/GdScO_3_ substrate: schematic polarization configuration (upper panel), a cross-section low-magnification TEM image (middle panel), and atomically resolved high-angle annular dark field scanning transmission electron microscopy (HAADF-STEM) images of two closure quadrant domains (bottom panel). Reproduced with permission [22]. Copyright 2015, The American Association for Advancement of Science. (b) Polar vortex lattice in SrTiO_3_/PbTiO_3_ superlattices on DyScO_3_ substrate: a schematic (upper panel), and a dark-field TEM image of a vortex lattice in (SrTiO_3_)_10_/(PbTiO_3_)_10_ (middle panel). Inset shows a magnified high-resolution scanning transmission electron microscopy (HR-STEM) image overlapping a polar displacement vector map for a vortex pair. A calculated phase diagram for (SrTiO_3_)_n_/(PbTiO_3_)_n_ illustrating the length scales within which different topological states can be stabilized (bottom panel). Reproduced with permission [23], Copyright 2016, Springer Nature; [78], Copyright 2017, American Chemical Society. (c) Polar skyrmion bubbles in a [(SrTiO_3_)_16_/(PbTiO_3_)_16_]_8_ superlattice on SrTiO_3_ substrate: schematic diagram (upper panel), a cross-section dark-field TEM image (middle panel), and a planar-view dark-field STEM image (bottom panel). Inset shows a skyrmion bubble configuration from calculations. Reproduced with permission [25]. Copyright 2019, Springer Nature.

Experimental evidence for polarization curling was not obtained until 2009 when Jia *et al.* [15] observed such a local structure at the junction between domain walls and substrate in PZT films, using aberration-corrected high-angle annular dark-field (HAADF) Z-contrast scanning transmission electron microscopy (STEM) that allows an atomic-scale visualization of local polarization. Nelson *et al.* also unveiled a closure-like domain texture near domain walls of BFO films. This texture only occurs close to insulating interfaces, indicating the critical role of depolarization energy [16]. It is also worth mentioning that these textures are not the classical four-fold quadrant domains with four 90° walls converging onto a central core, but rather considered as half-closure quadrants with walls converging at angles of 90° and 135^o^. In a recent seminal work, Tang *et al.* demonstrated a regular array of closure quadrant domains in PTO/STO superlattice deposited on a GdScO_3_ substrate, wherein each PTO layer (15–40 nm thick) is confined between two insulating STO layers, ensuring the poor screening condition of depolarization [22]. Along this line, Yadav *et al.* explored the (PTO)*_n_*/(STO)*_n_* superlattice on DyScO_3_ (DSO) substrate with thinner PTO layers (∼4 nm) and eventually achieved well-established vortex lattices (see Fig. 2b) [23], elucidating that size confinement is another ingredient for stabilizing the vortex domain structure. This effect was further supported by an observation of ultra-small polarization curling and closure domains (∼2 nm in diameter) in several unit-cell thick non-poled PTO ferroelectric tunneling junctions between Co and (La, Sr)MnO_3_ electrodes [24]. This demonstrates that smaller size (e.g. <10 unit cells) favors polarization curling even though the depolarization effect can be partially charge-compensated by the presence of electrodes.

To give a clear insight into the mechanism stabilizing these topological states, a combination of phase-field simulation, analytical discussion, and experimental observations was conducted on (PTO)*_n_*/(STO)*_n_* superlattice on DSO substrate [16,78]. It was revealed that a subtle competition between the electrostatic, elastic, and polarization gradient-related energy terms is involved over different length scales, resulting in thickness-dependent domain states [22,23]. More interestingly, a simplified quasi-2D analytical model based on the Landau–Ginzburg phenomenological theory predicted a narrow length-scale window for stabilizing the flux-closure vortex. The estimated upper critical scale for polarization curling and flux-closure vortex is one order of magnitude (actually ∼8 times) larger than the intrinsic domain wall width of bulk ferroelectrics. Since the domain wall for normal ferroelectrics should be a few lattice units wide, the critical scale for a flux-closure vortex should be on the order of ∼10 nm. The estimated lower threshold

of the critical length scale is on the same order of magnitude (∼1.4 times) as the intrinsic domain wall width. For PTO film, this lower limit (thickness) is ∼4.0 nm, below which the depolarization field is so strong that no vortex state can be stable, and an in-plane domain structure ensues.

It should be mentioned that this critical length-scale rule is basically determined by the competition between the depolarization energy and domain wall energy, and it provides a general estimation for the characteristic scale of a vortex or vortex-like structure in spatially confined systems. It can also serve as a simple intuitive design rule for searching for novel topological structures in ferroic systems, and provide a guide for designing and tuning vortex and other topological states.

Further explorations in similar ferroelectric superlattices led to more discoveries, including complex swirling topological feature and polar skyrmion bubbles that are akin to the intensively studied magnetic skyrmions, as done by Das *et al.*, in a similar (PTO)*_n_*/(STO)*_n_* (*n* ∼16) superlattice with a relatively large compressive strain from the STO substrate (see Fig. 2c) [25]. It was surprising to see that the polar structures display well-defined individual topological charge and chirality that can be macroscopically maintained, conforming the key feature of magnetic skyrmions. While polar skyrmions lack the chiral Dzyaloshinskii–Moriya interaction (DMI) that is necessary for stabilizing skyrmions, the underlying mechanism remains not well understood. The formation of such a skyrmion-like polar domain is in association with a specific type of bubble domain wall (loop wall) in nanoscale-confined ferroelectrics, wherein the local strong electric and stress fields not only reorient the polarization within the wall to form a ring but also rotate the polarization in adjacent domains surrounding the looped wall. Consequently, a polar bubble skyrmion pattern is generated.

It should be mentioned that the discoveries of chiral vortices and skyrmions in confined ferroelectric systems have activated research excitement in novel topological states that are akin to their magnetic counterparts but exhibit smaller length scales. These exotic states not only hold a promise for much denser and faster memory devices but also play an important role in modulating the overall physical behaviors of the ferroelectrics under investigation. These states could be harnessed to enhance the functionalities and performances of emergent structures and devices. A good example is the observation of enhanced negative capacitance behavior in association with vortex lattice patterns, which may be helpful for reducing the power consumption of transistors [79]. Additional questions and challenges are also posed, including how to individually switch, move, create, and erase these defects; what determines the switching energy, speed, and retention; and how to use these small defects for memory applications*.*

### Topological polar textures in nanodots/nanoislands

In parallel studies, polar topological domain structures in both vertically and laterally confined nanodots/islands have also been intensively explored, motivated by the substantial demand for high-density data devices. Such topological isolated nanostructures are compatible with high-density integration processing and also allow further electric control of individual topological domain states. In this subsection, we discuss the topological domains, particularly the closure domain/vortex and center domain observed in nanodots/islands [26–31,54–58].

#### Vortex and closure quadrant domains

**Figure 3.**
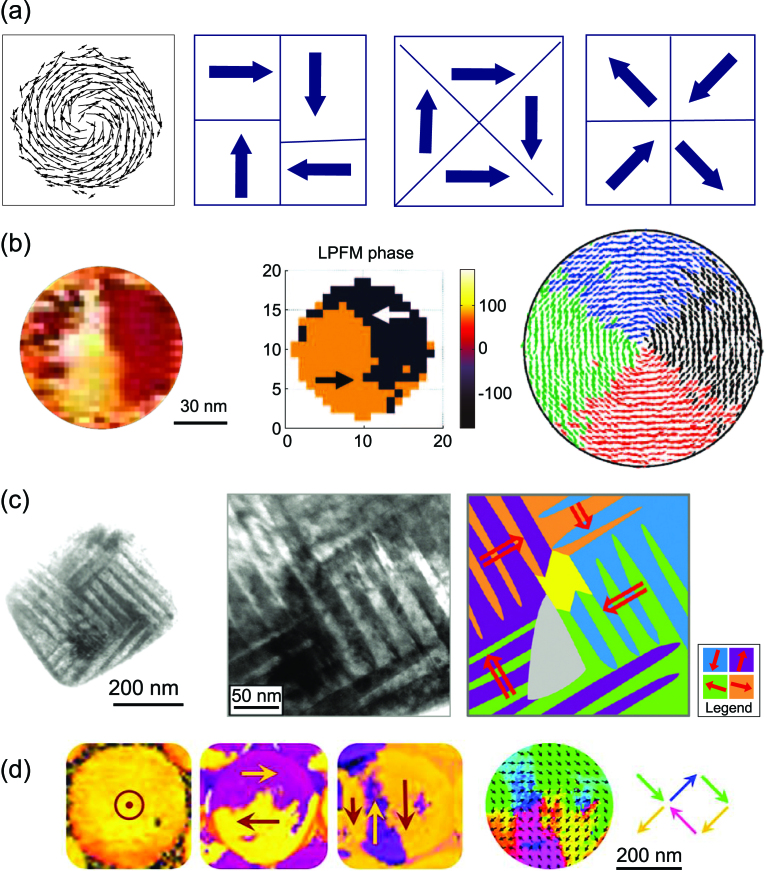
Some representative vortex and closure quadrant domains in ferroelectric and multiferroic nanoislands/nanoplates. (a) Polarization configurations for some topological domain structures: calculated polar vortex domain in ultra-small nanodots (reproduced with permission [14]. Copyright 2004, Springer Nature.), and schematics of closure quadrant, quadrant vortex, and quadrant anti-vortex domain states. (b) A vortex-like domain state in a Pb(Zr, Ti)O_3_ nanoisland on SrTiO_3_ substrate (∼60 nm in diameter) revealed by a combination of PFM observation and simulation. Reproduced with permission [55]. Copyright 2009, American Chemical Society. (c) A complex closure quadrant domain consisting of bundles of in-plane *a*_1_–*a*_2_ domain patterns in a free-standing Pb(Zr, Ti)O_3_ nanoplate (∼400 nm in lateral size) observed by TEM. Reproduced with permission [70]. Copyright 2011, American Chemical Society. (d) A quadrant vortex and anti-vortex pair in a BiFeO_3_ nanoisland (∼300 nm in diameter) on SrTiO_3_ substrate. Reproduced with permission [26]. Copyright 2017, American Chemical Society.

Earlier investigations of topological domain textures in low-dimensional nanodots were ignited by Naumov *et al.* [14] using the first-principles calculations and it was predicted that bi-stable polar vortices as small as 3.2 nm in diameter can be stabilized in highly depolarized ferroelectric nanodots. Such domain textures also eliminate the problem of crosstalk among neighboring nanodots, promising for ultrahigh-density memories with areal density of ∼60 Tbit/in^2^ (see Fig. 3a). Since then, a great number of theoretical and experimental efforts have been made in searching for stable vortices on various nanodots or nanoislands [57,58].

Earlier investigations did find experimental evidence of vortex or closure quadrant structures in confined systems. For example, vortex-like domain states were identified in high-density PZT nanodot arrays prepared by AAO template-assisted PLD [55] (see Fig. 3b). Unique closure quadrant or quadrant anti-vortex-like structures consisting of domain bundles were also observed by Schilling *et al.*, McGilly *et al.*, and McQuaid *et al.* in BaTiO_3_ and Pb(Zr, Ti)O_3_ single-crystal samples of micrometers in size (see Fig. 3c) [54,69,70]. Recently, the coexistence of quadrant vortex and anti-vortex domains in BFO nanoislands was demonstrated by Tian *et al.*, as shown in Fig. 3(d) [26].

However, unlike the regular array of vortex/closure quadrants in ultrathin films or superlattices, one can observe only sporadic closure quadrants rather than vortex states in very limited cases of nanodots, which does not conform well to theoretical predictions [57,59]. This inconsistency is probably attributed to the fact that the depolarization field is not sufficiently large to overcome the energy cost needed to accommodate the disclination strain from vortex domain formation or polarization rotation. It is also likely induced by the severe screening from the surface charge or point defects that relieves the depolarization strength in conjunction with the relatively large size of the structures under investigation. So far, a tough challenge remains to achieve a highly stable isolated vortex in ferroelectric nanodots/islands.

To achieve stable polar vortex or closure textures, one should improve the microstructural quality and dimension control of as-prepared nanostructures. It is also useful to choose those ferroelectrics with low-polarization–mechanical coupling (e.g. BFO or PZT) so that the disclination strain energy can be reduced [26,54]). Embedding ferroelectric nanodots into an insulating matrix to reduce charge screening could also be an effective strategy. Another strategy is to artificially create metastable vortex/closure states by applying external fields, as demonstrated by Balke *et al.* and Li *et al.* [17–19] where vortex-like domains in BFO films were created using the AFM probing tip.

#### Topological center domains

Recently another type of topological state, the so-called center domain, where all the polarization vectors point inward (or outward) to (from) the center core, was surprisingly identified in well-prepared multiferroic nanodots/nanoislands [27,28,31]. Such kinds of highly charged topological cores can be realized by properly controlling the fabrication procedure of nanodots. More interestingly, such polarization textures not only enable individual address coding and electric control but also exhibit intriguing domain wall conduction that holds great potential for memory applications with non-destructive readout of topological center-domain states [28].

**Figure 4.**
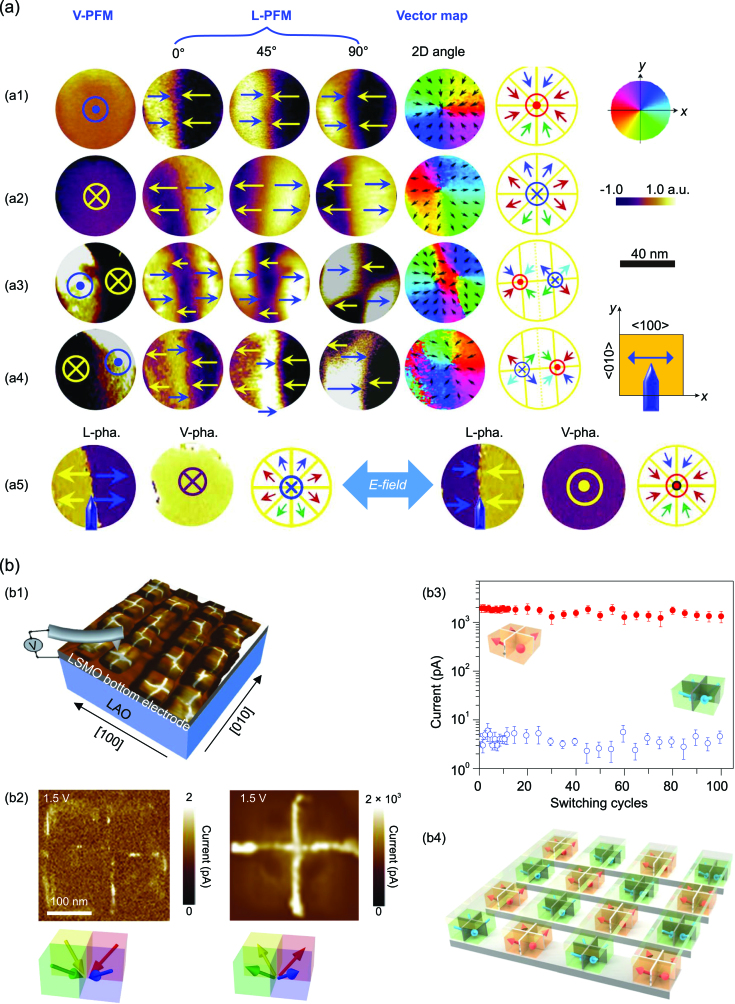
Topological center-domain and domain wall conduction in BiFeO_3_ nanoislands. (a) Four types of center-domain states in BiFeO_3_ nanodots on SrTiO_3_ substrate observed by vector PFM analysis: center-convergent (a1), center-divergent (a2), double-center domain with convergent and divergent center states (a3, a4), and panel (a5) illustrates a reversal switching between convergent and divergent center domains induced by an electric field. Reproduced with permission [27]. Copyright 2017, The American Association for Advancement of Science. (b) Switchable conductive domain wall states of quadrant center domains in square-shape BiFeO_3_ nanoislands on LaAlO_3_ substrate, and a conceptual prototype of memory devices based on the center domains: CAFM images of quadrant center domains in an array of nanoislands (b1), different conductive domain wall states for respective convergent and divergent domains (b2), endurance performance of resistive switching in domain wall conduction (b3), and a crossbar device architecture of conceptual memory prototype (b4). Reproduced with permission [28]. Copyright 2018, Springer Nature.

In 2017, Li *et al.* reported that a high percentage of center domains can be spontaneously formed in high-density arrays of small BFO nanodots [27]. As shown in Fig. 4a, a family of topological center domains such as center-convergent domains, center-divergent domains, and double-center domains were identified in BFO nanodots with a typical lateral size of ∼60 nm. A vector PFM analysis and polarization reconstruction scheme in comparison with phase-field simulations confirmed this special domain structure. More exciting is that these topological domains are rather robust yet can be reversibly switched between convergent and divergent center-domain states, as triggered by an electric field. This is a good merit for non-volatile memory applications.

The observation of such types of unique topological domains raises open questions, such as the mechanism for stabilizing such topological domains and further strategies to modulate and harness them for future applications. For the stability issue, one possible reason is that these domain textures can be stabilized by effective screening and inhomogeneous surface charge accumulation although they are supposed to carry large electrostatic energy, as revealed by experimental data and phase-field simulations [27]. Recently, quadrant center domains, accompanied with thin buffered domain structure, were observed in self-assembled square-shaped epitaxial BFO nanoplates (∼300 nm in thickness) embedded in CoFe_2_O_4_ (CFO) matrix by Kim *et al.*, and it was argued that the strain gradient in the nanoplates can be a reason for stabilizing these topological states [29–30]. Ma *et al.* observed reversible quadrant center domains in rhombohedral BFO nanoislands embedded in tetragonal phase matrix [28], and it was suggested that a combination effect from the tilted bottom edge, interfacial constraints from the matrix, and effective screening from charge carriers favors energetically the alignment of polarizations in parallel to the tilted bottom edges. A more recent study by Han *et al.* attributed the formation of quadrant center domains in isolated BFO nanoislands to the surface charge mechanism, whereby different surface charges and charged surface shapes can lead to dissimilar center-domain states [31]. It is also interesting to note that the high-resolution transmission electron microscopy (HRTEM) observations in this work revealed the existence of small Bi-deficient regions in the BFO nanoislands, providing a hint that point defects also contribute to the inhomogeneous accumulation of surface charges that stabilizes these center states.

Although there is still no agreement on whether the center domain is stabilized by strain- or charge-related mechanisms, observation of these unexpected domain states suggests the participation of additional physical ingredients like charges, point defects, and strain gradient, besides the usual depolarization energy and domain wall energy, in patterning the unusual topological domain structures. The above highlighted findings further exemplify that the dimension reduction in ferroelectric islands can provide an effective pathway to reach the individually addressable and controllable center domains, allowing further exploration of novel functionalities and application potentials. This perspective has been further evidenced recently by the remarkable features of cross-shaped domain wall conduction patterns associated with the quadrant center domains [28], a milestone step towards topological memory devices with non-destructive current readout of different topological states.

## EMERGENT PROPERTIES AND PROSPECTIVE APPLICATIONS OF TOPOLOGICAL DOMAINS

It is well established that many physical properties of ferroic materials are closely related to their domain structures. Therefore, these exotic domains in multiferroic nanostructures must offer new opportunities for exploring and tailoring nanoscale functionalities. For instance, domain walls, as a kind of 2D topological defect, actually provide ultra-narrow (e.g. a few atomic layers thick) quasi-2D objects that possess multi-functionalities (magnetic, electric, optical properties). These defects can be created, erased, and replaced by external fields, and thus can be considered as fundamental building blocks for future domain wall electronics [53,63].

Since the exciting discoveries of tantalizing domain wall conduction [80,81], a wide range of energy exotic properties in domain walls has been uncovered, including giant metallic conductivity in charged domain walls [82], magnetoresistance [83], photovoltaics [62], and resonant tunneling behaviors [84–87]. While so far most experimental and theoretical studies have focused on domain walls as 2D topological defects, modern integrated electronic devices require the creation and manipulation of quasi-1D objects that are more compatible with the current semiconductor technologies but nevertheless remain largely unexplored so far. With the emergence of various intriguing polar topological defects, more interest has been aroused in exploring exotic 1D topological defects and singularities. In this section, we will address this issue and readers may find more information on 2D domain walls in the literature [13].

Due to the ultra-small nature of 1D singularities, there exists a sudden change in polarization and strain state, resulting in a sizable variation of the local band gap and charge accumulation. Therefore, a distinct difference in the transportation and other physics behaviors between these singularities and the parent phase is expected. This difference was exemplified in 2012 by the intriguing finding of Balke *et al.*, who first demonstrated the controllable creation of 1D topological defects in BFO films and revealed a local enhancement in conductivity in small vortex/anti-vortex cores [17]. The existence of local and ultra-small conductive channels was explained by an external-bias-driven transition from the vortex core to a metastable twist structure that is similar to a segment of charged domain wall and thus of high conductivity. It can also be understood by a dynamic conductor generated from the coupled response of polarization, electrons, and mobile vacancies under external electric bias for reading. Such conductive 1D channels also allow further control by external fields, suggesting a pathway for design and implementation of high-density integrated devices. Unfortunately, the observed conductive current in the vortex core is rather small, on the order of 10^−12^ A, probably inappropriate for constructing nanoscale electronic devices.

A recent experiment on another type of topological domains, the so-called center-type domains in self-assembled square-shape BFO nanoislands [28], demonstrated a significant enhancement of conductivity. As shown in Fig. 4b, a distinct cross-shaped high conductive pattern inside each individual island was observed by conductive atomic force microscopy (CAFM), which is closely related to the highly conductive charged domain wall in a center-type domain. It was also found that the tail-to-tail charged domain walls in the center-divergent states (probably *p*-type BFO) exhibit much higher conductance than those in the head-to-head charged domain walls in the center-convergent states. This enables the non-destructive readout of high-conductivity center-divergent and low-conductivity center-convergent states. The controlled switching of the two different conductive domain states becomes accessible and switch cycling up to 100 cycles with a sizable on/off resistance ratio over 10^3^ was realized.

The distinct difference in the conductive level between the upward and downward polarization states is somewhat similar to the case of ferroelectric resistive switching. Nevertheless, it is more likely related to the switching between different types of charged domain walls, e.g. the switching between the head-to-head and tail-to-tail walls. As suggested earlier, the existence of charged domain walls is associated with a significant band bending and an accumulation of electrons and holes, accounting for the dramatic change in conductivity [82]. In the present cases, the tail-to-tail wall tends to increase the valence band and attract holes in the *p*-type BFO, contributing to the significantly enhanced conductivity. In contrast, the head-to-head wall tends to attract point defects (e.g. Bi vacancies) and thus no sizable change in conductivity can be observed. One important advantage of this type of topological center domain is the deterministic switch between the head-to-head and tail-to-tail walls, a good merit for memory devices inaccessible in normal head-to-tail walls. Based on these findings, a prototype memory device was proposed by adopting a crossbar architecture, where electrode lines cross an array of individual conductive domain walls confined in an array of nanoislands.

While these observations provide the possibility of programming various conductive states by controllable switching between these topologically protected states, various programmable conductive domain wall patterns in BFO cramped nanoplates have been attempted by writing up different center-domain patterns [30]. This attempt does open routes towards further design of configurable devices, based on programmable wall conduction states in individually addressable nanoislands of center-domain structure. This approach is compatible with the semiconductor technology for high-density integration devices. What should be mentioned here is that the high conduction states observed in these center domains are mainly contributed by the conductive charged domain walls rather than the topological defect cores. Thus, an open question remains in terms of the mechanism for the conductive core of the center-domain structure.

The discoveries of unusual conduction features in these topologically non-trivial domain walls also spur further explorations of other exotic physical phenomena originating from 1D topological defects. One example is the finding of tantalizing negative capacitance behaviors when a change in charge distribution causes the opposite change in net voltage across the material. This finding is in association with the vortex lattices in PTO/STO superlattice structures [79]. The observed negative capacitance is mainly from domain walls that process high energy and large polarizability, confirmed by a combination of direct TEM observation and local electric field reconstruction. These behaviors may be harnessed to reduce the supply voltage requirement in a transistor where the sub-threshold swing can be improved, thus making computers and other electronic devices more energy efficient. These phenomena also elucidate the critical role of such collective topological defects in defining the macroscopic properties of the materials under investigation. Similarly, open questions remain in terms of the availability of negative capacitance in materials with other types of defects (e.g. skyrmions) and how to tune this property via external fields or domain engineering.

It should be mentioned that investigations into emergent properties and functionalities of 1D topological defects are still in the early stages, and further explorations in this fertile field will surely bring about more discoveries and application opportunities. One can expect unusual findings in terms of magnetism, magnetoresistance, photovoltaics, memristive, and electro-optic effects of these defects. Surely, these prospected novel functionalities may provide excess fundamental building blocks for tunable high-performance topological electronic devices, such as memories, local magnetic field or strain sensors, energy-efficient transistors, actuators, optoelectronics, and piezoelectronic devices. There are also some open questions, e.g. whether the complex 1D defects display similar or dissimilar properties to the 2D domain walls, and what the theoretical framework is that can accurately predict the microscopic or macroscopic properties of these topological defects.

## ELECTRIC-FIELD-DRIVEN MAGNETIC SWITCHING

Among those claimed functionalities of multiferroics, the ME effect is the core effect, enabling the mutual control of magnetic and electric properties, in particular the intriguing electric-field-driven magnetic switching (EDMS) that holds promise for high-density, high-speed, and energy-efficient spintronic devices [12,88–90]. For a typical MeRAM unit, the estimated energy consumption to switch the magnetization can be as low as ∼10^−18 ^J per bit (in a 10 nm × 10 nm lateral geometry), four orders lower than that of the state-of-the-art spin torque approach (10^−15 ^J per bit [44]). To fulfill the requirement for the next generation of devices, robust, repeatable, and pure electrically driven magnetization switching at room temperature is urgently needed. In addition, the devices must be scalable down to the nanoscale. Certainly, the size and interfacial effects in multiferroic nanostructures could provide a new degree of freedom to manipulate domain structure [34,38–40] and ME coupling [5], and thus offer alternative pathways to realizing EDMS functionality. In this section, we only present a brief overview of the ME properties in nanostructured multiferroic heterostructures, particularly emphasizing the effect of domain geometry on EDMS behaviors, while more comprehensive overviews are available in the literatures [8–10].

### Size and interface effects

The ME effect in nanostructured multiferroics differs from that in bulk and thin films, owing to the effective release of the clamping effect from substrates and film geometry, increased interfaces/surfaces, and the remarkable change of domain structures [32–33]. It has been reported that self-assembled column-composites (1–3 type coupling) exhibit much higher ME eco-efficiency, compared to that of multilayer (2–2 type coupling) architecture [9], owing to the partial release of substrate clamping and more interfaces between the two phases. This enhanced ME effect enables electric switching of magnetic domains with or without the assistance of external magnetic fields. The well-ordered arrangement of such self-assembled columns can be further improved using the seed-assisted method, as shown by Stratulat *et al.* [91]. Recently, Tian *et al.* synthesized a high-density array of BFO/CFO heterostructured nanodots (0–0 type coupling) with lateral size ∼60 nm, and the local ME coupling was probed using magnetic force microscopy (MFM) and piezoresponse measurement [33]. A significantly enhanced ME coefficient, enabling both magnetic control of piezoelectric properties and electric switching of magnetic domains, was obtained. However, magnetic switching triggered by electric field is not controllable in the reported 0–0 type systems, seriously inhibiting further device applications.

### Improving the controllability of EDMS by tuning magnetic domains

It is well known that the magnetic domain structure of nanomagnets is greatly determined by the geometric parameter due to the prominent shape anisotropy at the nanoscale [92–94]. This property provides a new degree of freedom to manipulate and improve the controllability of EDMS in nanostructured multiferroic heterostructures. For instance, repeatable full 180° switching of a single nano-domain via a procession switching scheme triggered by an ultrafast electric pulse in Ni nanomagnets deposited on Pb(Mg_1/3_Nb_2/3_)O_3_-–PbTiO_3_ (PMN–PT) substrates (0–2 type multiferroic heterostructures) has been predicted [38–40]. A full 180° domain reversal can also be accomplished by two successive and deterministic 90° switches in a multiferroic heterostructure consisting of a patterned flower-shaped nanomagnet (Ni) with a four-fold magnetic axis on PMN–PT substrate [38]. Other ‘peanut’ and ‘cat-eye’ shaped nanomagnets on PMN–PT substrates allow similar 180° magnetization rotations [39].

**Figure 5.**
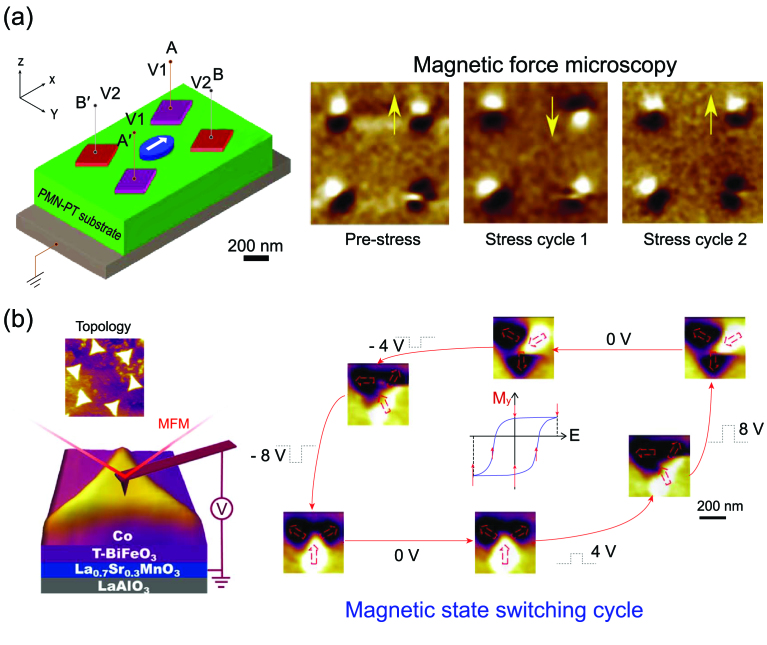
Two examples illustrating the improved switchability for electric-field-driven magnetic switching via tuning magnetic domains. (a) Electric-field-driven 180° magnetization reversal process in elliptical Co nanomagnets on piezoelectric Pb(Mg_1/3_Nb_2/3_)O_3_–PbTiO_3_ substrate: schematic device structure (left panel), and MFM images illustrating a back-and-forth 180° magnetization reversal in one of the four Co elliptical nanomagnets induced by two sets of consequential electric pulses, wherein the arrows indicate the magnetic directions of the switchable nanomagnet (right panel). Reproduced with permission [41]. Copyright 2017, American Chemical Society. (b) Electric-field-driven reversible 120° magnetic state rotation in triangular-shape Co nanomagnets on tetragonal-structured BiFeO_3_ film on LaAlO_3_ substrate: schematic of the multiferroic heterostructure and experimental set-up, and MFM images illustrating the evolution of domain states in a nanomagnet as a function of a set of sequential electric pulses, which is analogous to a hysteresis loop with magnetization (*M*) versus electric field (*E*) (see inset). Reproduced with permission [34]. Copyright 2018, American Chemical Society.

Nevertheless, these approaches either require an accurate control of ultrafast time or complicated lithographic processes, restricting them from experimental practice. Recently, Biswas *et al.* developed an effective two-step switching strategy and realized the electrically driven deterministic 180° reversal of magnetization in isolated elliptical Co nanomagnets at room temperature [41]. As shown in Fig. 5a, electric field was applied via two pairs of electrodes on a PMN–PT substrate, wherein the lines joining the centers of the electrode pairs intersect the major axis of the ellipse nanomagnet at ∼ +30° or −30°. After applying two consequential electric pulses from two different electrode pairs that produces two consequent magnetoelastic anisotropy pulses parallel to the electric fields, the single-domained nano-ellipse undergoes two successive rotations for 120° and then relaxes to the opposite 180° direction, completing the reversible switching. Unfortunately, due to the inhomogeneities in ferroelectric domain switching and interfacial pinning, only a small portion of the magnetic nanomagnets can be reversibly switched.

As PMN–PT-based multiferroic heterostructures usually require high driving voltages (∼100 V), a drawback for nano-device applications, EDMS in other systems was also attempted. Yao *et al.* [34] demonstrated a reversible 120° magnetic state rotation driven by an electric field in the Co/BFO heterostructures, as shown in Fig. 5b. The heterostructure consists of a triangular Co nanomagnet array on a tetragonal-BFO (T-BFO) film deposited on LaAlO_3_ (LAO) substrate. A 120° magnetic state rotation of the Co nanomagnet, triggered by a set of voltage pulses, was realized, evidencing the highly appreciated magnetization–electric field (*M*–*E*) hysteresis. Moreover, the switching can be triggered by an ultrafast (∼10 ns) electric pulse with a good switch cycling performance. Such reversible switching is interpreted as a combination of shape-anisotropy-assisted interface strain, exchange coupling between the BFO film and Co nanomagnets, and the shape anisotropy of the triangular Co nanomagnets, as supported by the micromagnetic simulations. The strain-mediated coupling mainly originates from the phase transition between the tetragonal phase and the rhombohedral phase in the BFO film. This strain is transferred to the top Co nanomagnet, switching the easy axis of magneto-anisotropy. The exchange coupling between the BFO film and Co nanomagnet is able to break the time-reversal symmetry through a sequence of procession switching, as suggested by Heron *et al.* [95]. In addition, the shape anisotropy guarantees switching only among the thermodynamic stable states.

In short, it is suggested that domain engineering for multiferroic heterostructures by adjusting the geometric parameters is an effective way to control the switching and improve the switching controllability. Nevertheless, so far, the switching repeatability remains rather low. To improve the controllability and repeatability, topologically protected magnetic domains such as vortices and skyrmions may be adopted, as demonstrated by a recent experiment where electric field creation and annihilation of skyrmions were reported [43,96].

### EDMS modulation via polar domain engineering

Besides the size effect, interfacial coupling, and magnetic domain, the EDMS can be also modulated by polar domain structure via the dynamic switching sequence. One may call this strategy the domain engineering of EDMS. One may take again multiferroic BFO thin films as examples. It is known that the antiferromagnetic (AFM) order and polarization are coupled intrinsically via the Dzyaloshinskii–Moriya (DM) effect, wherein the DM vector, which has the same direction as the net magnetization moment (**M_c_**) from the canted antiparallel Fe^3+^ spins, the AFM vector (**L**), and the ferroelectric polarization (**P**) are strongly coupled and orthogonal to each other [97]. Once a thin soft magnetic layer (e.g. CoFe) is placed on the underlying BFO film, exchange coupling between the DM vector and magnetization of the magnetic layer allows electric control of the magnetic states of the magnetic layer by controlling the DM vector [95].

**Figure 6.**
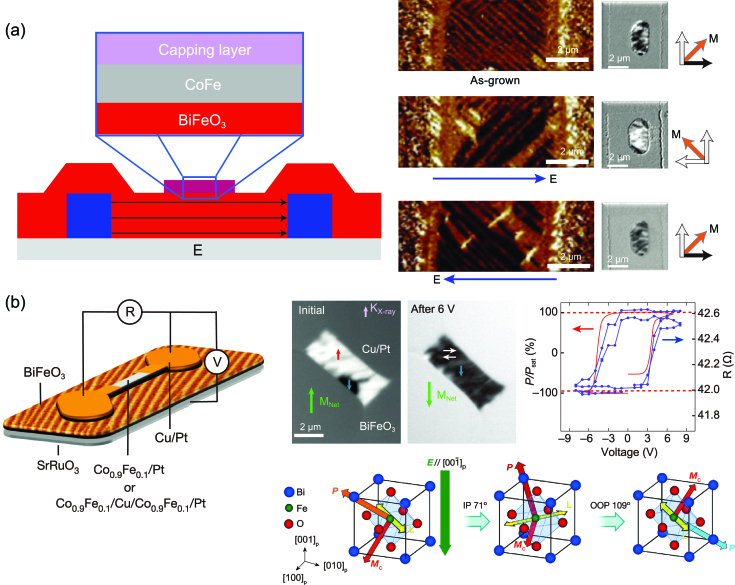
Two examples evidencing the modulation of electric-field-driven magnetic switching by tuning the polarization switching in CoFe/BFO heterostructures. (a) A 90° net magnetic switching in CoFe magnet triggered by a 90° net in-plane polarization switching (71° ferroelastic switching) of BiFeO_3_ induced by in-plane electric fields: a schematic device structure (left panel), and the switching of both ferroelectric stripe domain pattern (PFM images) and net magnetic domains (X-ray magnetic circular dichroism photoemission electron microscopy (XMCD-PEEM) images) triggered by electric fields (right panel). Reproduced with permission [35]. Copyright 2008, Springer Nature. (b) Deterministic 180° magnetic switching triggered by 180° polarization switching using an out-of-plane electric field: schematic of the heterostructure (left), and XMCD-PEEM images for the 180° switching of net magnetic domain before and after applying a voltage, along with the multi-cycle hysteresis loops of the magnetoresistance of a spin valve induced by an electric field (upper right panel); schematics illustrating the mechanism of a 180° procession (two-step) polarization switching that induces coherent switching of the DM vector and net magnetic moment Mc in BiFeO_3_ film (bottom of the right panel). Reproduced with permission [95]. Copyright 2014, Springer Nature.

Along this line, Chu *et al.* [35] reported pure electric-field-driven magnetic switching in a micrometer-sized CoFe magnet deposited on a epitaxial BFO film on SrTiO_3_ substrates, whereby a 90° net magnetization switching of CoFe magnets was triggered by a 71° ferroelastic switching (90° switching on in-plane projection) of net polarization for a stripe-domained BFO thin film (see Fig. 6a). Due to the internal and interfacial coupling, polarization **P** is always perpendicular to the directions of the **L** and DM vectors, and thus a 90° switching of the in-plane projected net polarization leads to a coherent 90° switching of net magnetization in the CoFe magnet.

In contrast, a net 180° in-plane polarization switching (net 109° ferroelastic switching) of a stripe-domained pattern for a BFO film deposited on DyScO_3_ (DSO) substrate enables the 180° switching of net magnetization in the CoFe magnet on the top of BFO film [36]. The net 180° switching of in-plane polarization is a result from the two variants switching. One variant undergoes a 90° clockwise rotation (71° ferroelastic switching) while the other rotates 90° anticlockwise, thus producing an overall net 180° switching of the in-plane polarization. Accordingly, different switching directions of the two variants lead to different coherent 90° rotations of sub-domains in the CoFe magnet and eventually an overall 180° net magnetization switching.

It is noted that the two examples highlighted above used different underlying substrates. One is STO and the other is DSO, and the deposited BFO films thus exhibit quite different polar domain structures. Consequently, different strain and clamping states with the BFO films were generated. More interesting is that a deterministic and pure 180° net magnetization reversal can be trigged by the 180° polarization procession switching via a perpendicular electric field (see Fig. 6b) [95]. This is a breakthrough towards EDMS-based and energy-efficient MeRAM devices [98,99]. This is rather unexpected because a pure 180° polarization switching is believed not to change the direction of the antiferromagnetic axis and the **DM** vector. Magnetic switching should not be possible. This unusual consequence can be understood by the fact that the 180° polarization switching actually consists of two consequent ferroelastic switching steps, and accordingly two-step procession switching leads to two-step coherent switching of the **DM** vector and eventually the pure 180° net magnetization switching. This consequence was revealed by the fast PFM observations and first-principles calculations and an electrical switch of magnetoresistance in a spin-valve device becomes possible, a milestone step towards nanoscale, low-energy-consumption, non-volatile magnetoelectronics.

Finally, we present a brief discussion of an emergent effect observed in 0–0 type heterostructured nanoislands in which domain engineering may also be utilized to tune the EDMS behavior. It is noted that distinctly different polarization switching behaviors will be expected once a ferroelectric film is patterned into small nanoislands, due to the release of clamping effects from the substrate and surrounding domains. For instance, it was found that a perpendicular electric field drives the BFO thin film to undergo a ferroelastic 109° polarization switching, followed by a relaxation to the 180° direction. This observation does not work for small BFO nanoislands where only a single ferroelastic switching occurs [100,101]. Therefore, much faster switching speed of EDMS and enhanced thermal stability of the switched magnetic states can be obtained in CoFe/BFO nanoislands, as compared with those in CoFe nanomagnets/BFO film (0–2 type) [101]. This is a nice case to illustrate the substantial impact of specific domain structure in nanoferroics. In fact, extraordinary in-plane piezostrain as large as ∼1.0% was predicted in PZT nanoislands via the 90° polarization rotation, favorable for improving the EDMS performance in multiferroic nanostructures [102].

## SUMMARY AND PROSPECTS

Multiferroic nanostructures possess a wealth of exotic domain structures, in particular rich topological states (summarized in Table 1), and thus provide an ideal playground for a plethora of physic properties and functionalities that might underpin a wide range of future electronic devices. It is intriguing that domain engineering provides an effective way to tailor the ME effect. This field has emerged as a topic of broad interest while it is still in its infancy. One is thus allowed to present some questions and prospective new directions to be explored.

### Mechanism for on-demand topological defects necessary for device realization is not established

The design and controlled manipulation of polar topological domains is a critical issue for exploring novel physics and device applications. This requires a comprehensive and accurate understanding of the formation mechanism of various domain states. Previous theoretic frameworks, as proposed by Naumov *et al.* [14], Kornev *et al.* [77], and Chen *et al.* [57], can explain the formation of vortex and skyrmion textures in PTO/STO superlattices [23,25,78], and also provide a clear understanding of the length-scale and boundary conditions for different topological states. While these theories do not work well for interpreting why the center domains are more stable than the vortex/closure domains in some nanostructures by considering the involvement of carriers and charged defects [27–29], and substantial efforts are needed to comprehend the complicated competition between various interactions and energy terms accounting for the intermixing of point defects, carriers, strain, and external fields.

### Tuning polar topological domains

The switching of topological domains is an intriguing aspect deserving close attention and it intimately links the materials’ functionalities with the performance of reconfigurable nanoscale devices. Controllable switching and programming of center-type domains have been demonstrated, accompanied with significant change of conduction [28]. Optical illumination-induced switching of topological states was also observed [103], offering possibilities of new device paradigms, e.g. voltage-free optical writing memory devices. It is now strongly believed that the whole packet of physical properties of multiferroics relies critically on the dynamic or static behaviors of polarization and domain structure. For instance, the local conduction of the vortex core is related to the dynamic twist state [17], and vortex-related negative capacitance is related to the dynamic displacement of local dipoles [79]. An exploration of these dynamic and static behaviors subjected to various stimuli may open an entirely new avenue to a wealth of emergent properties and functionalities. Surely, open questions remain along this line. Is there any coupling between magnetic topological states and polar states? What are the responses of these states in response to multi-fold stimuli other than an electric field?

### Switching magnetic topological domains

Electric-field-driven magnetic switching holds promise for energy-efficient MeRAM and other spintronic devices, and key challenge is to realize high performance EDMS at nanoscale. The topologically protected exotic domains may provide a new pathway to achieve this goal, as exemplified by electric field creation and annihilation of magnetic skyrmion states [96]. Besides, magnetic skyrmion also allows injection by light [104], further extending its application possibility. It is intriguing that the nucleation and annihilation of skyrmion do not involve the breaking of time-reversal symmetry that is required in convention magnetization reversal. In this sense, electric field triggering is much easier than the magnetic field operation. These topological states also have potential to scale down to few nanometers. Successful tuning of these states may push forward the realization of denser, faster, and energy-efficient MeRAMs and other spintronic devices. To achieve this goal, new methods for a non-volatile and deterministic switch of the magnetic states as well as a convenient readout of different topological states are urgently needed.

### Advanced characterization techniques

The push to accurately understand domain structures and relevant functionalities exerts challenges on characterization techniques. The recent advances in advanced HRTEM and scanning probe microscopy (SPM) have enabled the observation of polar domains at atomic or nanometer scales [27,28], while magnetic domains can be imaged by Lorenz force TEM (LTEM) [96], photoemission electron microscopy (PEEM) [95] or magneto-optical Kerr (MOKE) microscopy. Recently, 3D

mapping of vortices in ferroelectric nanoparticles was obtained using the X-ray-based Bragg coherent diffractive imaging technique [105]. However, each individual technique has its own limitations and it is tough to probe *in situ* the domain structure and its dynamics against various stimuli. It is also particularly challenging for probing nanoscopic and multi-functional cross-coupling behaviors.

**Figure 7.**
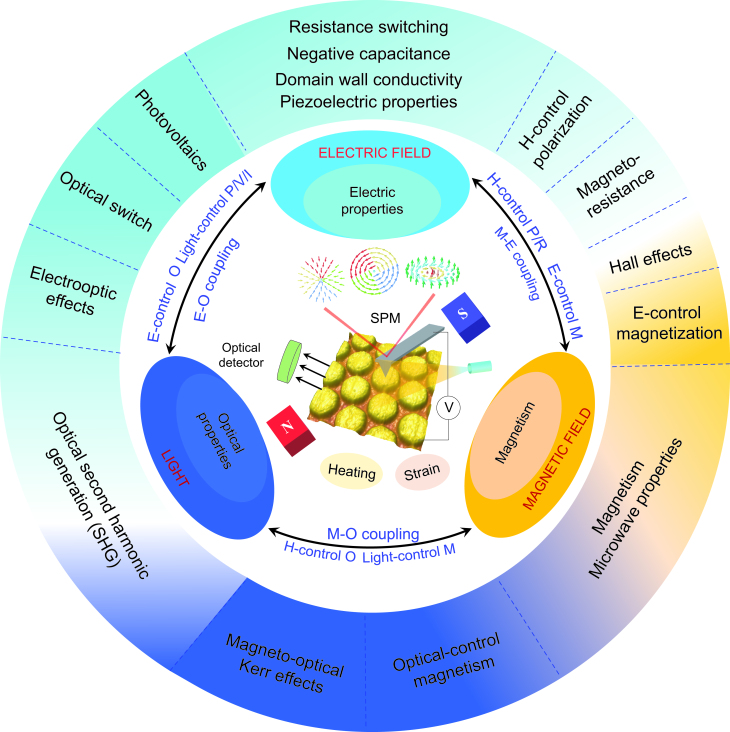
A sketched experimental system based on scanning probe microscopy that enables the probing and tailoring of multi-functionalities and properties under multi-fields/stimuli, and a highlight of possible functionalities/properties in multiferroic nanostructures that could be characterized by this system.

Nevertheless, it is worth spending some time on the multi-functional SPM technique as a versatile and powerful nanoscale probe of multi-functionalities. Nowadays, multi-field stimuli, like electric field, magnetic field, light illumination, strain field, and thermal field, are able to be integrated with the advanced SPM technique. Figure 7 shows a schematic of the SPM characterization platform that not only enables the *in situ* monitoring of domain structure and its dynamics, but also probing its related functional response at the nanoscale, including domain wall conductivity, memrestive, photovoltaics, and ME response etc. Furthermore, it is expected that tip-enhanced (or near-field) optical probing in Raman, electro-optic, and MOKE modes can also be incorporated in the SPM system. Certainly, spatial and temporal resolution should be given the top priority for all these advanced probing functionalities.

### Prospective device applications

Although there are still limited examples demonstrating the potential applications of these topological states, the intriguing properties that have been revealed are sufficient for us to posit some possible devices. High-density memory (or logic) devices could be one application terminal for the topological domains (vortex, skyrmion), offering high density, fast switching, and energy-efficient read/writing. Along this line, deterministic control of the topological domains and enhanced switching performances (creation, annihilation, speed, retention, endurance) is needed. In addition, accurate and efficient readout of different states can be a critical issue, whereby non-destructive readout is highly appreciated [28]. Other choices may include the capacitance sensing, near-field optical sensing, and ME probing. The data write/read, triggered by light illumination, mechanical strain, or magnetic field, may lead to other types of memory, such as optical write/current read, or full optical memory.

Another issue is the integration of material structures with current semiconductor technologies, and the isolated nanoislands may show some advantages over the crossbar architectures. Polar skyrmions could be applied in track record memory, while capabilities to inject and drive the skyrmions are needed. Other prospective applications, like transistors based on negative capacitors, strain/magnetic/optical sensors, and microwave devices based on domain resonance, can also be expected.

Last but not least, it should be pointed out that most of the results reviewed in this article were obtained based on TEM and SPM observations, and this leaves a gap between the microscopic findings and practical device applications. Bridging this gap requires further efforts.
